# Book Reviews

**Published:** 1908-10

**Authors:** 


### BOOK REVIEWS

---

PROGRESSIVE MEDICINE, VOL. III, SEPTEMBER, 1908. A Quarterly Digest of Advances, Discoveries and Improvements in the Medical and Surgical Sciences. Edited by Hobart Amory Hare, M. D., Professor of Therapeutics and Materia Medicine in the Jefferson Medical College of Philadelphia. Octavo, 287 pages, with 30 engravings. Per annum, in four cloth-bound volumes, $9.00; in paper binding, $6.00, carriage paid to any address. Lea & Febiger, Publishers, Philadelphia and New York.

The September volume of Progressive Medicine deals helpfully with four topics of great practical importance.

Under the title of Diseases of the Thorax and its Viscera, Professor Ewart gives an admirable summary of the recent advances in our knowledge of tuberculosis. The treatment of emphyema, the associated disturbances which may arise in disorders of the heart, blood pressure and its relation to disease are topics with which the author has dealt in a manner which will command special attention.

Dr. Gottheil's contribution, covering Dermatology and Syphilis, possesses much of interest, even for those whose practice lies outside the lines of these subjects. In particular, we would allude to his views as to the use of carbon dioxide in the treatment of nevi and other growths and to the sections devoted to the uses and limitations of the x-rays in diseases of the skin. The general resume devoted to Syphilis is excellent.

The advance in our knowledge of Obstetrics has been very completely covered by Dr. Davis. Among the topics in his contribution, which possesses more than ordinary interest, may be mentioned changes in the various organs of the body produced by pregnancy, eclampsia, ectopic gestation, face presentation, narcosis during labor, pubiotomy and the management of the third stage of labor.

The department of Nervous Diseases concludes the volume. The author, Dr. William G. Spiller, has produced a very complete and lucid review of the advances in this rather abstruse department of medicine, as is especially apparent in his treatment of the subject of brain tumors and locomotor ataxia.

Progressive Medicine occupies a field apart from that of the magazine. It performs for the general practitioner, the surgeon and the specialist a most important service, bringing him knowledge which he could not otherwise obtain, either by his own efforts or in any other publication.

Most of the advances in medicine are of course first announced in periodicals as the quickest means of publicity. Many of them are lost to the man who does not read a half dozen languages, and this vital knowledge would moreover be limited to very small circles were it not for the existence of this medium for universal diffusion.

---

ANATOMY, DESCRIPTIVE AND SURGICAL. By Henry Gray, F. R. S., late lecturer on Anatomy at St. George's Hospital, London. New American edition, enlarged and

